# Development and characterization of 30 microsatellite loci for *Plagiorhegma dubium* (Berberidaceae)

**DOI:** 10.1002/aps3.1200

**Published:** 2018-12-04

**Authors:** Bo‐Yun Kim, Soo‐Rang Lee, Yong‐In Kim, Dae‐Hyun Kang, Young‐Dong Kim

**Affiliations:** ^1^ Multidisciplinary Genome Institute Hallym University Chuncheon 24252 Republic of Korea; ^2^ International Biological Material Research Center Korea Research Institute of Bioscience and Biotechnology Daejeon 34141 Republic of Korea; ^3^ Department of Life Science Hallym University Chuncheon 24252 Republic of Korea

**Keywords:** Berberidaceae, EST‐SSR marker, genetic diversity, medicinal plant, microsatellites, *Plagiorhegma dubium*

## Abstract

**Premise of the Study:**

*Plagiorhegma dubium* (Berberidaceae) has been listed as an endangered species in Korea due to extensive collection and destruction of natural habitats. In this study, 30 microsatellite loci, including 25 polymorphic loci, were developed for *P. dubium* for use in population‐level genetic analyses.

**Methods and Results:**

We carried out transcriptome sequencing and isolated a total of 30 expressed sequence tag–simple sequence repeat markers from *P. dubium* using Illumina HiSeq high‐throughput sequencing. To test utility of the developed markers, we genotyped 60 individuals from three populations and estimated the number of alleles and levels of observed and expected heterozygosity. Expected heterozygosity levels ranged from 0.000 to 0.594, 0.000 to 1.000, and 0.000 to 0.744 in the three populations, respectively.

**Conclusions:**

These transcriptome‐derived simple sequence repeat markers are highly polymorphic and can be widely used in characterization of the endangered *P. dubium*. Population genetic studies with these markers will provide valuable insights for conservation by unraveling evolutionary patterns of *P. dubium*.

The genus *Plagiorhegma* Maxim. (Berberidaceae) is composed of only one species, *P. dubium* Maxim., which has a narrow distribution in northeastern Asia and Siberia (Jeong and Sivanesan, 2016). *Plagiorhegma dubium* has been listed as an endangered species by the Ministry of the Environment of South Korea due to its rarity and specific habitat preference (Kim, 2006; Ghimire and Heo, 2012). The plants have a long history of medicinal uses: roots have long been used as folk medicine for stomachache, and the phytochemicals extracted from the plants have shown remedial effects on elevated cholesterol levels (Kong et al., 2004). *Plagiorhegma dubium* is also appreciated as an ornamental plant for commercial use due to its showy flowers and heart‐shaped leaves (Huang, 1995). Despite growing interest in the species in both the pharmaceutical industry and horticulture, there is a complete lack of knowledge on the *P. dubium* genome. No applicable microsatellite markers have been developed for genetic studies and genetic analyses at the population level, which can provide insights for conservation and management plans of rare and threatened species (Ottewell et al., 2016). Simple sequence repeats (SSRs), also known as microsatellites, are the most frequently used molecular markers due to their abundance, codominant mode of inheritance, and multiallelic and highly polymorphic nature (Lopez et al., 2015). However, the development of microsatellite markers is an expensive and time‐consuming processes (Squirrell et al., 2003). Expressed sequence tag (EST)–derived SSRs can overcome some of the drawbacks of older methods, for example by decreasing processing time (Zhou et al., 2016). Here, we developed and characterized 30 EST‐SSR markers for the rare and threatened *P. dubium* using transcriptome sequencing with the Illumina paired‐end sequencing platform. We evaluated the performance of these markers using 60 individuals representing three populations of *P. dubium*.

## METHODS AND RESULTS

### Transcriptome sequencing

To prepare a cDNA library, total RNA of *P. dubium* was extracted from a fresh leaf of a single sample from Korea (voucher no. NIBRVP0000556155; Appendix 1). RNA was extracted using the RNeasy Kit version 2.2 (Illumina, San Diego, California, USA) following the manufacturer's instructions, and was used for TruSeq cDNA library preparation. The RNA libraries were sequenced on the Illumina HiSeq 2000 platform, producing 150‐bp paired‐end reads. All raw reads were submitted to the National Center for Biotechnology Information (NCBI) Sequence Read Archive (Bioproject ID PRJNA472226). Trimmomatic 0.32 (Bolger et al., 2014) was used to remove adapters and low‐quality reads with the following parameters: seed mismatch of 2, palindrome clip threshold of 30, simple clip threshold of 10, a minimum adapter length of 2, headcrop of 7, leading and trailing quality of 3, sliding window size of 4, with an average quality of 20 and a minimum sequence length of 50 bases. After trimming, reads were assembled into 94,785 transcripts using the short‐read assembly program Trinity (Haas et al., 2013) with default settings; these were then clustered into 76,725 unigenes.

### Development of microsatellite markers based on ESTs of *P. dubium*

Microsatellites from the unigenes were detected using MISA version 1.0.0 (Thiel et al., 2003) with the default parameters. The criteria for identifying SSRs were as follows: the minimum number of nucleotide repeats was six for mono‐, di‐, tri‐, tetra‐ penta‐, and hexanucleotides. MISA identified 11,458 SSRs, of which 96 primer pairs were selected for further testing based on (1) region containing at least five repetitions of di‐ or trinucleotide motifs; (2) PCR product size of 140–500 bp; (3) length ranging from 12 to 24 nucleotides; (4) annealing temperature 55–60°C; and (5) minimum GC content 50%. Primer pairs were designed using Primer3 (Rozen and Skaletsky, 1999) to flank the microsatellite‐rich region with a minimum of six repeats.

The utility of the selected 96 microsatellite markers was evaluated by PCR on three individuals from each population of *P. dubium*. Reactions were carried out in a total volume of 25 μL containing 2.5 μL of 10× Ex Taq buffer (TaKaRa Bio Inc., Shiga, Japan), 2 μL of 2.5 mM dNTPs, 0.1 μM of forward and reverse primer, 0.1 μL of TaKaRa Ex Taq, and 5–10 ng of template DNA. All PCRs were performed in a GeneAmp PCR System 9700 thermocycler (Applied Biosystems, Carlsbad, California, USA) using the following protocol: initial denaturation at 98°C for 5 min; followed by 30 cycles of denaturation at 95°C for 1 min, annealing at locus‐specific annealing temperature (Table 1) for 1 min, and extension at 72°C for 1.5 min; and a final extension step at 72°C for 10 min. Of the 96 candidate markers, 34 markers amplified and were suitable for further testing. Finally, 30 markers were selected and included in the following analysis after excluding four markers with a low amplification rate of 80% or less. The PCR products were labeled with fluorescent dyes (HEX and FAM) and run on an ABI 3730XL automated sequencer (Applied Biosystems) using the GeneScan 500 LIZ Size Standard (Applied Biosystems). Genotyping was manually determined with GeneMapper 3.7 (Applied Biosystems). Identified microsatellite markers (Table 1) producing clear and polymorphic bands were subsequently used for genetic diversity assessments.

**Table 1:** Characteristics of 30 microsatellite loci developed in *Plagiorhegma dubium* and tested for this study

Locus	Primer sequence (5′–3′)	Repeat motif	Allele size range (bp)	Fluorescent dye	*T*_a_ (°C)	GenBank accession no.	Putative function [Organism]	*E*‐value
JD_03^a^	F: CATCGCCACCTCAATCCTCA	(TG)_7_	247	HEX	60	Pr032816664	Not found	–
	R: GCAGATCCGATCCCAGTCAA							
JD_05	F: TCGGCAGAAATGGAGCACTT	(TG)_9_	273–277	FAM	60	Pr032816654	Not found	–
	R: GGGTGTGGGTGTCTGACAAT							
JD_11	F: AGACTTGGGAGGTAACCAAACC	(TA)_9_	196–198	FAM	60	Pr032816640	Not found	–
	R: TCTCCCCTTTGGCTTGCATT							
JD_14^a^	F: TTGGGTGTCTACAGCAGCTG	(TA)_6_	248	FAM	60	Pr032816662	Hypothetical protein PRUPE_ppa020282mg [*Prunus persica*]	4E‐130
	R: GCTTGTGGCCCCTAGAGTTT							
JD_17^b^	F: GTGTGTACCCATCCCATCCC	(GT)_6_	184–188	FAM	55	Pr032816636	Predicted: transcription elongation factor B polypeptide 2‐like isoform X2 [*Citrus sinensis*]	1E‐52
	R: TGGTGTGAGTTGGTAAGCCA							
JD_19	F: TGATTCGCCAACCCCTTCAA	(GT)_10_	262–266	FAM	60	Pr032816656	Hypothetical protein MTR_8g085190 [*Medicago truncatula*]	1E‐56
	R: GCTTTGAGACTGTCCCGGAA							
JD_24^b^	F: GCATGATTGCTGCTCCTGTG	(GA)_10_	222–226	HEX	55	Pr032816650	Mitogen‐activated protein kinase kinase 5 [*Petroselinum crispum*]	2E‐159
	R: CGTTTGCTGTGGAGAGAGGT							
JD_27^b^	F: TCTGCAAAATTGGGTGCTGC	(CT)_7_	165–170	FAM	60	Pr032816655	Unnamed protein product [*Vitis vinifera*]	0.0
	R: GCATTGTCATGGCTAGCTGC							
JD_29	F: ACTTCAGCTAGCAGTGCGTT	(CT)_10_	176–180	FAM	60	Pr032816660	Predicted: E3 ubiquitin‐protein ligase UPL3‐like isoform 1 [*Vitis vinifera*]	0.0
	R: TCAACGACTCAACCTGCCTC							
JD_30^a^	F: ATCTGCTGTTGCTGCTGTCT	(CA)_9_	198	FAM	55	Pr032816651	R2R3‐MYB transcription factor MYB9 [*Epimedium sagittatum*]	3E‐126
	R: AGACGACAGCTTTTACCGCA							
JD_33	F: CTTGCCCCTATGTAGCTCCG	(CA)_6_	204–212	FAM	60	Pr032816663	Not found	–
	R: ACGATGGTGCAGATGTGGTT							
JD_35^b^	F: AGTCGGCCCAGAATTTGGTT	(AT)_8_	266–268	FAM	55	Pr032816657	Not found	–
	R: TTGCAGCACGTTGGTCTTTG							
JD_36^b^	F: TGCTGCTCCACTACCTTGTG	(AT)_6_	191–193	FAM	60	Pr032816658	Predicted: E3 ubiquitin‐protein ligase At1g63170 [*Prunus mume*]	6E‐142
	R: TCCATTAGCAGCGGGTGTTT							
JD_38	F: GCAATGACCATGGACATCGC	(AT)_11_	182–184	FAM	60	Pr032816661	Hypothetical protein JCGZ_15866 [*Jatropha curcas*]	8E‐125
	R: ACTGCCTGACAAGCGATGAA							
JD_42^b^	F: CTGATCAAGACTCCCGCCTC	(AG)_6_	142–144	HEX	55	Pr032816645	Hypothetical protein PRUPE_ppa005757mg [*Prunus persica*]	0.0
	R: CGAGGTGGTAATCTCGGCTC							
JD_43^b^	F: TTTGTGGCCAATTGCTGTGG	(AG)_6_	480–488	HEX	55	Pr032816652	Predicted: uncharacterized membrane protein At3g27390‐like [*Solanum tuberosum*]	2E‐121
	R: AACGTGGAAGGCATGAACCT							
JD_50	F: GCCCCATCTATCCTCTGCAC	(TTC)_6_	275–278	HEX	55	Pr032816659	Unnamed protein product [*Vitis vinifera*]	9E‐194
	R: ACACGACACCCATTACCACC							
JD_52^b^	F: AGTGCTAAGAACACGGTGGG	(TTC)_6_	218–220	HEX	55	Pr032816637	Predicted: uncharacterized protein LOC100261915 [*Vitis vinifera*]	9E‐158
	R: TCACGACGCCCTATCATGTG							
JD_54^a^	F: ACGCAACAATCGCGAAAACA	(TGG)_6_	183	FAM	60	Pr032816639	Not found	–
	R: ACCCTCATCCCGACTCTCAA							
JD_66	F: CCCCAGCCCTACCAATAACC	(GCA)_7_	215–224	HEX	60	Pr032816644	Unnamed protein product [*Vitis vinifera*]	4E‐108
	R: CGAACAGTACAGAGGCCACA							
JD_68	F: TCCCACCACCTACCCTTCTT	(GAG)_8_	282–294	FAM	60	Pr032816647	Hypothetical protein POPTR_0010s13850g [*Populus trichocarpa*]	4E‐101
	R: AGGTCTTGTCCCCGATCTCA							
JD_71^a^	F: GCAGCAACAACTCCAACCAA	(GAA)_7_	266	FAM	60	Pr032816646	Predicted: transcriptional regulator ATRX homolog [*Prunus mume*]	1E‐28
	R: GTTCCTGTGCAACAATGGCA							
JD_72^b^	F: GCGGACTGTTCTGAAGTGGA	(CTT)_7_	259–262	FAM	60	Pr032816642	Not found	–
	R: ACATAGCTGCACCGAGACAA							
JD_77	F: CTCGGCTAACTGGACGGTAC	(CGG)_6_	180–186	FAM	60	Pr032816641	Predicted: zinc finger A20 and AN1 domain‐containing stress‐associated protein 4 isoform 1 [*Vitis vinifera*]	2E‐59
	R: CTGTTCCGGGTATCGATGCA							
JD_78	F: TGATGAGGCTTGTGCACCAT	(CGC)_6_	200–203	FAM	55	Pr032816653	Unnamed protein product [*Vitis vinifera*]	1E‐168
	R: TGCAGGTTCAAATGGGTCGA							
JD_79^b^	F: AGACGGTGGCAATGAATGGT	(CCT)_6_	220–223	HEX	60	Pr032816638	Decarboxylating‐like 6‐phosphogluconate dehydrogenase [*Medicago truncatula*]	0.0
	R: TAAGTCACACAAGGCCCGTC							
JD_81	F: CCTGCCTTTTCCCAAACTGC	(CCA)_6_	223–229	HEX	55	Pr032816649	RNA‐binding CRS1/YhbY domain‐containing protein, putative [*Theobroma cacao*]	0.0
	R: CCAAGGCCGAGGCATAGTAG							
JD_82	F: GCCCTCGGAGTTTTTGGAGA	(CAT)_6_	254–257	FAM	55	Pr032816643	Not found	–
	R: GCTCCGGCTGCATGAAATAG							
JD_83^b^	F: AAGGAGCACAGCGACATGAA	(CAG)_6_	216–219	FAM	60	Pr032816648	GATA transcription factor 9 [*Morus notabilis*]	5E‐61
	R: GGGCATAGGTGGTGGGAAAA							
JD_93	F: TCACTCAGCACCGCTCATTT	(ACA)_7_	234–247	HEX	55	Pr032816635	Hypothetical protein AMTR_s00029p00127080 [*Amborella trichopoda*]	3E‐25
	R: CCCGGCTGCGTAATTGATTG							

Note

*T*_a_ = annealing temperature.

a Monomorphic loci.

b Fixed heterozygotes.

### Genetic parameters

Fresh leaves from 60 *P. dubium* individuals were sampled from three populations from Korea, Japan, and China. The voucher specimens were deposited in the National Institute of Biological Resources Herbarium (KB), Incheon, Republic of Korea (Appendix 1). The precise locations of the sites have been withheld to prevent illegal collection. The level of polymorphism at each locus was assessed by calculating the number of alleles per marker (*A*), observed heterozygosity, and expected heterozygosity using GenAlEx 6.5 (Peakall and Smouse, 2012). Deviation from Hardy–Weinberg equilibrium was estimated with Arlequin 3.5 (Excoffier and Lischer, 2010).

Functional annotations for these 30 markers were compared against the NCBI nonredundant (NR) protein database with BLASTX (*E*‐value 1 × 10^−5^). A total of 30 markers were successfully amplified, of which polymorphism was detected in 25 (Table 1). Because 11 markers (17, 24, 27, 35, 36, 42, 43, 52, 72, 79, and 83) were fixed for heterozygotes in all 60 samples, we present genetic parameters only for the remaining 14 markers (Table 2).

**Table 2:** Genetic diversity in three *Plagiorhegma dubium* populations based on 14 newly developed polymorphic microsatellite markers.^a^

Locus	Korea (*n* = 20)			China (*n* = 20)			Russia (*n* = 20)		
	*A*	*H*_o_	*H*_e_ b	*A*	*H*_o_	*H*_e_ b	*A*	*H*_o_	*H*_e_ b
JD_05	3	1.000	0.584^**^	2	1.000	0.500^**^	3	1.000	0.525^**^
JD_11	2	0.684	0.450^*^	2	0.100	0.095	2	0.950	0.499^**^
JD_19	3	1.000	0.594^**^	3	0.950	0.559^**^	3	1.000	0.605^**^
JD_27	2	0.400	0.320	1	0.000	0.000	2	0.050	0.139^**^
JD_33	2	1.000	0.500^**^	2	1.000	1.000^**^	3	1.000	0.623^**^
JD_38	2	0.250	0.289	2	0.850	0.499^**^	2	0.400	0.455
JD_50	2	1.000	0.500^**^	2	0.400	0.375	2	0.313	0.264
JD_66	1	0.000	0.000	1	0.000	0.000	3	0.200	0.580^**^
JD_68	2	1.000	0.500^**^	4	1.000	0.590	4	1.000	0.744^**^
JD_77	2	0.050	0.469^**^	1	0.000	0.000	3	0.100	0.185^**^
JD_78	2	0.263	0.411	2	0.200	0.180	1	0.000	0.000
JD_81	2	0.050	0.049	1	0.000	0.000	2	0.050	0.139^**^
JD_82	2	0.250	0.219	2	1.000	0.500^**^	2	0.950	0.499^**^
JD_93	2	0.950	0.499^**^	2	0.950	0.499^**^	2	1.000	0.500^**^
Mean	2.071	0.564	0.384	1.929	0.532	0.307	2.429	0.572	0.411

Note

*A* = number of alleles; *H*_e_ = expected heterozygosity; *H*_o_ = observed heterozygosity; *n* = number of individuals.

a Locality and voucher information are provided in Appendix 1.

b Significant deviation from Hardy–Weinberg equilibrium after correction for multiple tests (*P < 0.05 and **P < 0.01).

The number of alleles per locus was estimated from 14 polymorphic EST‐SSR markers among three populations of *P. dubium* (*A* = 1–4, average = 2.143; Table 2). Levels of observed heterozygosity for each locus ranged from 0.000 to 1.000. Levels of expected heterozygosity ranged from 0.000 to 0.594, 0.000 to 1.000, and 0.000 to 0.744 in the three sampled populations (Table 2). No significant linkage disequilibrium was found in all pairs of 30 loci after Bonferroni correction (α = 0.05), whereas some markers revealed significant deviation from Hardy–Weinberg equilibrium (Table 2).

## CONCLUSIONS

We developed and amplified a set of novel microsatellite markers for *P. dubium*. These markers will be used for constructing an in situ and ex situ conservation strategy of the species by estimating the level of genetic diversity and population structure in wild populations. Assessment of its genetic variation could contribute to inferences of the past evolutionary history of *P. dubium*. Furthermore, of the 11,458 SSR loci identified, additional markers could be developed to address research needs.

## DATA ACCESSIBILITY

Raw reads were submitted to the National Center for Biotechnology Information (NCBI) Sequence Read Archive (Bioproject ID PRJNA472226). Sequence information for the developed primers has been deposited to NCBI; GenBank accession numbers are provided in Table 1.

## APPENDIX 1 Locality and voucher information for *Plagiorhegma dubium* populations sampled in this study.^a^,^b^

**Table:**

Population	Locality	*N*	Voucher no.
Korea	Uiseong, Gyeongbuk	20	NIBRVP0000556155
China	Yanbian, Jilin	20	NIBRVP0000601483
Russia	Vladivostok, Primorskiy	20	NIBRVP0000556157

Notes

*N* = number of individuals.

a Voucher specimens were deposited in the Herbarium of the National Institute of Biological Resources (KB), Incheon, Republic of Korea.

b Precise locations of the collection sites have been withheld to prevent illegal collection.
